# Different Features of Tumor-Associated NK Cells in Patients With Low-Grade or High-Grade Peritoneal Carcinomatosis

**DOI:** 10.3389/fimmu.2019.01963

**Published:** 2019-08-21

**Authors:** Silvia Pesce, Valerio Belgrano, Marco Greppi, Simona Carlomagno, Margherita Squillario, Annalisa Barla, Mariella Della Chiesa, Stefano Di Domenico, Domenico Mavilio, Lorenzo Moretta, Simona Candiani, Simona Sivori, Franco De Cian, Emanuela Marcenaro

**Affiliations:** ^1^Department of Experimental Medicine, University of Genoa, Genoa, Italy; ^2^Department of Surgical Sciences and Integrated Diagnostics, IRCCS Policlinico San Martino, University General Hospital, University of Genoa, Genoa, Italy; ^3^Department of Surgery, Institute of Clinical Sciences, Sahlgrenska Academy at the University of Gothenburg, Sahlgrenska University Hospital, Gothenburg, Sweden; ^4^Centre of Excellence for Biomedical Research, University of Genoa, Genoa, Italy; ^5^Department of Informatic Bioengineering, Robotic and System Engineering, University of Genoa, Genoa, Italy; ^6^Unit of Clinical and Experimental Immunology, Humanitas Clinical and Research Center, Rozzano, Milan, Italy; ^7^Department of Medical Biotechnologies and Translational Medicine, University of Milan, Milan, Italy; ^8^Department of Immunology, IRCCS Bambino Gesù Children's Hospital, Rome, Italy; ^9^Department of Earth Science, Environment and Life, University of Genoa, Genoa, Italy

**Keywords:** human NK cells, peritoneal carcinomatosis, pseudomyxoma peritonei, NK cell receptors, immune escape, immune checkpoint, PD-1/PD-L, NKp30

## Abstract

Peritoneal carcinomatosis (PC) is a rare disease defined as diffused implantation of neoplastic cells in the peritoneal cavity. This clinical picture occurs during the evolution of peritoneal tumors, and it is the main cause of morbidity and mortality of patients affected by these pathologies, though cytoreductive surgery with heated intra-peritoneal chemotherapy (CRS/HIPEC) is yielding promising results. In the present study, we evaluated whether the tumor microenvironment of low-grade and high-grade PC could affect the phenotypic and functional features and thus the anti-tumor potential of NK cells. We show that while in the peritoneal fluid (PF) of low-grade PC most CD56dim NK cells show a relatively immature phenotype (NKG2A+KIR–CD57–CD16dim), in the PF of high-grade PC NK cells are, in large majority, mature (CD56dimKIR+CD57+CD16bright). Furthermore, in low-grade PC, PF-NK cells are characterized by a sharp down-regulation of some activating receptors, primarily NKp30 and DNAM-1, while, in high-grade PC, PF-NK cells display a higher expression of the PD-1 inhibitory checkpoint. The compromised phenotype observed in low-grade PC patients corresponds to a functional impairment. On the other hand, in the high-grade PC patients PF-NK cells show much more important defects that only partially reflect the compromised phenotype detected. These data suggest that the PC microenvironment may contribute to tumor escape from immune surveillance by inducing different NK cell impaired features leading to altered anti-tumor activity. Notably, after CRS/HIPEC treatment, the altered NK cell phenotype of a patient with a low-grade disease and favorable prognosis was reverted to a normal one. Our present data offer a clue for the development of new immunotherapeutic strategies capable of restoring the NK-mediated anti-tumor responses in association with the CRS/HIPEC treatment to increase the effectiveness of the current therapy.

## Introduction

Peritoneal carcinomatosis (PC) is a histologically heterogeneous, progressive malignant extremely rare disease. It is characterized by the accumulation of tumor tissue in the peritoneal cavity that ranges in biologic behavior from benign to highly malignant (1–4).

Low-grade peritoneal diseases include well-differentiated papillary mesothelioma, a rare subtype of epithelioid mesothelioma, arising from the mesothelial layers of the peritoneum and pseudomyxoma peritonei, a mucinous adenocarcinoma of the appendix spread to the peritoneal cavity which is characterized by recurrent voluminous mucinous ascites (4). These tumors grow along the peritoneal surfaces (feature that makes them susceptible to surgical debulking) (1). Although frequently characterized by an indolent course, they are not a harmless condition, and are fatal if untreated. A second category of peritoneal diseases includes peritoneal mesothelioma, an aggressive malignancy arising from mesothelial cells within the serosal lining of the peritoneum, and peritoneal mucinous carcinomatosis, an invasive, high-grade, poorly differentiated carcinoma with large extracellular pools of mucin. This second type originates from mucinous carcinomas of the gastrointestinal tract (5), gallbladder (6), pancreas, or ovary (7), thus representing the metastatic spread of a primary cancer resulting in peritoneal carcinomatosis. Similar to high-grade peritoneal mesotheliomas, it is always associated with poor patients survival.

In view of the poor efficacy of classical therapies, alternative treatments have increasingly been applied, including the cytoreductive surgery (CRS) associated with intraoperative intraperitoneal chemohyperthermia (HIPEC) (1, 8, 9). In general, in low-grade histologic variants this treatment gives excellent long-lasting results, while responses in high-grade peritoneal mucinous carcinomatosis are limited. Currently, there is no marker that allows to predict which patients may benefit from these aggressive treatments. Moreover, the cellular and molecular mechanisms responsible for the proliferative potential and the resistance to therapy by peritoneal carcinomatosis tumor cells have not been elucidated. In this context, however, it became clear that immunological alterations occurring in the tumor microenvironment seem to favor tumor growth and implantation, thus resulting in a poorer prognosis. Indeed, PC tumor cells can develop different mechanisms of immune escape capable of suppressing the activity of the immune system, including induction of PD-1 expression on T cells and release of pro-tumoral/immunosuppressive cytokines (such as IL6, IL10, and TGF-beta1) that may either regulate tumor growth or modify the anti-tumor immune responses (10–12).

Natural Killer (NK) cells play a fundamental role in the immune response against tumor cells (13, 14). For this reason, immunotherapies exploiting anti-tumor NK cell activity are the most promising in the treatment of so far incurable tumors (15–20). NK cell functions (21), including potent anti-tumor cytolytic activity, cytokine production, and cross-talk with different immune cells (22), are controlled by a balance of several inhibitory and activating receptors (13, 23). In humans, the inhibitory receptors include HLA-I-specific and non-HLA-I specific receptors. The HLA-I-specific inhibitory receptors are represented by the well-known killer immunoglobulin-like receptors (KIR) that recognize the polymorphic HLA-A, -B, and -C molecules, the immunoglobulin-like receptor 1 (LIR-1/ILT-2) that is specific for different HLA-class I molecules, and the CD94/NKG2A heterodimer that recognizes HLA-E, a non-polymorphic non-classical HLA molecule (24–26). The non-HLA-I-specific inhibitory receptors include the immune checkpoint PD-1 that was originally described on T cells (27), but recently shown also on a subset of peripheral blood (PB) NK cells from healthy HCMV+ individuals and in tumor patients (28–30). The PD-1+ NK cell subset is mainly composed of fully mature cells, expressing the CD56dimKIR+LIR−1+NKG2A–CD57+CD16bright surface phenotype (29). The main non-HLA-I-specific activating NK receptors are NKp46, NKp30, NKp44 (called “natural cytotoxicity receptors,” NCR), NKG2D, and DNAM-1 (31, 32). Many of the activating NK receptor ligands have been identified and shown to be variably expressed by tumors (14).

Given the importance of NK cells in anti-tumor responses, it is not surprising that tumors use a wide range of mechanisms to avoid recognition by NK cells (33). Many mechanisms have been described, such as expression of tumor ligands for inhibitory receptors, induction/up-regulation of inhibitory receptors expression by NK cells, release of soluble ligands for activating NK receptors, downregulation of activating receptors on NK cells (19, 28, 34–36), and release of pro-tumoral/immunosuppressive soluble factors. These mechanisms lead to a suppression of anti-tumor NK cell activity and to an uncontrolled tumor growth.

In the present study, we analyzed PB and peritoneal fluid (PF)/ascites derived NK cells in patients with low-grade or high-grade PC and the ability of the PC tumor microenvironment to shape the NK cell compartment. We show that both low-grade and high-grade PC patients show an impaired NK cell phenotype, but with defects of different nature (e.g., downregulation of the main activating NK cell receptors vs. up-regulation/induction of inhibitory receptors such as PD-1). In addition, we observed different functional impairments in low-grade and high-grade PC patients. This analysis indicates that, based on the tumor grade, PC microenvironment may differently contribute to the tumor escape from immune surveillance.

Notably, after CRS/HIPEC treatment, the altered NK cell phenotype of a patient with a low-grade disease and favorable prognosis was reverted to a normal one (37).

Although in this study we examined a small group of patients, especially because of the rarity of this disease, our observations represent an important basis to carry on further investigations and to better understand the mechanisms underlying the development of PC and the possible intervention increasing the effectiveness of the CRS/HIPEC treatment by restoring also the NK-mediated anti-tumor responses.

## Methods

### Patients and Samples

This study included 8 patients with peritoneal carcinomatosis (PC) who had tumor surgery on PC between 2015 and 2018:

– 2 patients with well-differentiated papillary mesothelioma of the peritoneum (Pt. 6, Pt. 7)
– 3 patients with low-grade pseudomyxoma peritonei (Pt. 1, Pt. 8, Pt. 9)
– 2 patients with mucinous peritoneal carcinoma arising from epithelial ovarian cancer (Pt. 2, Pt. 3)
– 1 patient with mucinous PC arising from colorectal cancer (Pt. 4)

Samples of PB and PF/ascites from all the patients were collected before CRS/HIPEC treatment (time 0) and, when possible, at different time points after treatment (time 1: 48 h, time 2: 7 days) and analyzed without further processing or culturing with the exception of primary cell lines and functional experiments (see below).

Mononuclear cells from heparinized PB and from PF were obtained by density gradient centrifugation over Ficoll (Sigma, St. Louis, MO), and then resuspended in RPMI 1640 medium, supplemented with 2 mM glutamine, 50 μg/mL penicillin, 50 μg/mL streptomycin, and 10% heat-inactivated FCS (Fetal Calf Serum, Biochrom Ltd). Buffy-coats (healthy controls) were collected from volunteer blood donors admitted at the blood transfusion center of IRCCS Ospedale Policlinico San Martino, Genova, Italy.

In some cases, we obtained primary cell lines by culturing PF free cells for 2 weeks in the presence of PF derived from the matched patients.

### Ethical Statements

This study was carried out in accordance with the recommendations of the ethical standards of the institutional and/or national research committee. The protocol was approved by the ethics committee of the Liguria Region, Genova, Italy (no. 428Reg2014 for PC patients and no. 39/2012 for healthy donors). All subjects gave written informed consent in accordance with the Declaration of Helsinki.

### Monoclonal Antibodies

The following mAbs generated in the Laboratory of Molecular Immunology, DIMES, University of Genoa were used in this study:

A6/136 (IgM, anti-HLA-1); L14 (IgG2A, anti-Nectin-2); 5A10 (IgG1, anti-PVR); 11PB6 (IgG1, anti- KIR2DL1/S1); GL183 (IgG1, anti-KIR2DL2/L3/S2); AZ158 (IGg2A, anti-KIR3DL1/S1/L2); C227 (IgG1, anti-CD69); BAB281 (IgG1, anti- NKp46); AZ20 (IgG1, anti-NKp30); QA79 (IgG1, anti-p75); F278 (IgG1 anti-LIR1); ECM217 (IgG2b, anti-NKG2D); GN18 (IgG3, anti-DNAM-1); PP97 (IgM, anti-CD45).

The following purchased mAbs were used in this study: anti-ULBP1 (clone M295), anti-ULBP2 (clone M310), anti-ULBP3 (clone M550) (Amgen, Seattle, WA); anti-CXCR4-APC (R&D Systems, MN, USA); anti-ESA, IgG1 (Novocastra Laboratories Ltd.); anti-CD90, IgG1 (BD Biosciences, Pharmingen, CA, USA); anti-CD16-PerCP5.5 (clone 3G8) (BD Biosciences, Pharmingen, CA, USA); anti-CD107-PE (clone H4A3) (BD Biosciences, Pharmingen, CA, USA); anti-NKG2A-APC (clone Z199) (Beckman Coulter/Immunotech, Marseille, France); anti-PD-1 PE (clone 1.3.1.3) (Miltenyi Biotec, Bergisch Gladbach, Germany); anti-CD57-Vioblue (Miltenyi Biotec, Bergisch Gladbach, Germany); anti-B7-H6 (clone MAB7144) (R&D Systems, MN, USA); anti-PD-L1 (clone 27A2) (MBL, Woburn, MA); anti-PD-L2 (clone 176611) (R&D Systems, MN, USA); anti-CK7 (clone OV-TL 12/30) (Abcam, Cambridge, Regno Unito); anti-MUC2 (clone Ccp58) (Abcam, Cambridge, Regno Unito); anti-CD56-PC7 (clone c218) (Beckman Coulter/Immunotech, Marseille, France); anti-CD3-Viogreen (BW264/56 clone), anti-CD19-VioGreen (LT20 clone), anti-CD14-Viogreen (TÜK4 clone) (Miltenyi Biotec, Bergisch Gladbach, Germany).

### Flow Cytometer Analysis

Analyses were performed using a FACSCalibur or a FACSVerse flow cytometer (BD) and data were analyzed using the CellQuestPro software or the FacsSuite software, respectively (Becton Dickinson, Mountain View, CA). We also used FlowJo v10 for visualization of the unbiased t-distributed stochastic neighbor-embedding (t-SNE) algorithm. Analysis of NK cells was made on CD56dimCD3-CD19-CD16+/– gated cells. For KIR analysis, we used a pool of anti-KIR2DL1/S1, anti-KIR2DL2/L3/S2, and anti-KIR3DL1/S1/L2 mAbs. For analysis of CD45neg primary cell lines derived from PF cells, we analyzed the surface expression of different markers (including CD90, ESA, CK7, MUC2) and a series of ligands for NK cell receptors (including HLA-I, PVR, Nectin-2, B7-H6, ULBPs, PD-L1, and PD-L2 molecules) (38). In degranulation experiments, performed with frozen cells, the 7AAD (BD Biosciences, Pharmingen, CA, USA) nucleic acid dye was used for the exclusion of non-viable cells in flow cytofluorimetric assays.

### Cytokine Production

To analyze the presence of a panel of different soluble factors, we used two different kits (Bio-Plex Pro Human Cytokine 48-plex Assay, Bio-Plex Pro Human TGF-beta1 and TGF-beta2 Assay from Bio-Rad) (Hercules, CA, USA) and all assays were performed by Bioclarma s.r.l. (Torino, Italy) according to Biorad kit procedures. Soluble B7-H6 (sB7-H6) was measured in cell supernatants by enzyme-linked immunosorbent assay (Human B7-H6 DuoSet ELISA, R&D Systems, MN, USA).

### Cell Line and Degranulation Assays

The target cell line used in this study was the human erythroleukemia K562 cell line.

For degranulation assay, PB and PF lymphocytes were cultured O.N. in the presence of sub-doses of IL15 (0.5 ng/ml) and then co-incubated with K562 target cells at an E/T ratio of 1:4 in a final volume of 200 μl in round-bottomed 96-well plates at 37°C and 5% CO_2_ for 3 h in culture medium supplemented with anti-CD107a-PE mAb. Surface staining was done by incubating the cells with anti-CD3, anti-CD56 anti-CD19, and anti-CD16 for 30 min at 4°C. The cells were washed and analyzed by flow cytometry (FACSVerse, Becton Dickinson). Analysis of NK cells was made on CD56+ CD3− CD19− gated cells.

### Statistical Analysis

The unsupervised hierarchical clustering was performed by using the online tool MORPHEUS (https://software.broadinstitute.org/morpheus/).

In order to compare the expression of the three groups simultaneously (i.e., HD-NK, PB-NK, and PF-NK), we computed the Krustall rank sum tests for each cell surface marker analyzed.

**Figure 1:**
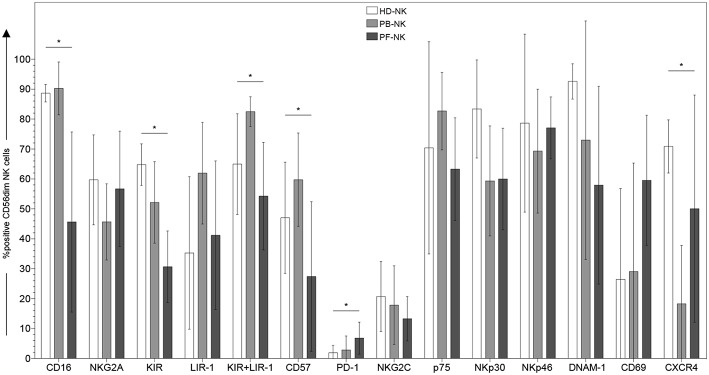
Surface phenotype of CD56dim NK cells derived from peripheral blood or peritoneal fluid of patients affected by PC. Cytofluorimetric analysis of the expression of different cell surface markers on CD56dim-gated NK cells from peripheral blood of healthy donors (HD-NK) (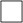 bars), peripheral blood of PC patients (PB-NK) (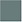 bars) (*n* = 8: Pt. 1, Pt. 2, Pt. 3, Pt. 4, Pt. 6, Pt. 7, Pt. 8, Pt. 9) and peritoneal fluid of PC patients (PF-NK) (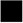 bars) (*n* = 6: Pt. 1, Pt. 2, Pt. 3, Pt. 4, Pt. 6, Pt. 8). Histograms indicate the percent ±SD of HD-/PB-/PF-NK cells positive for the indicated receptors. In order to compare the expression of the three groups simultaneously (i.e., HD-NK, PB-NK, and PF-NK), we computed the Krustall rank sum tests for each cell surface marker analyzed. ^*^P < 0.05.

**Figure 2:**
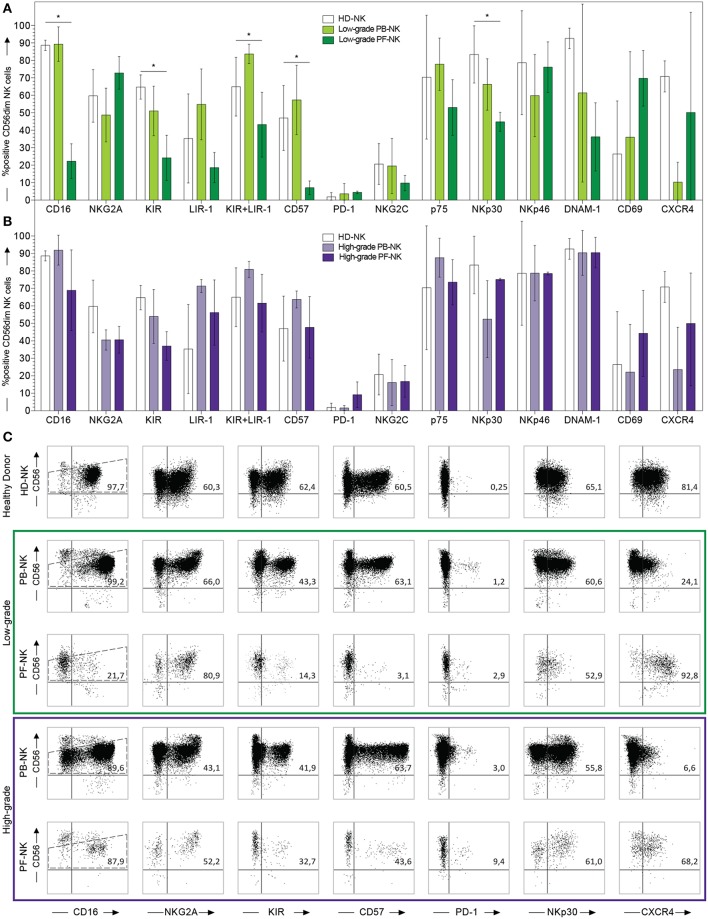
Comparison between PB- and PF-NK cells derived from low-grade and high-grade PC patients. Cytofluorimetric analysis of the expression of a panel of cell surface markers on HD-NK (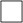 bars) (*n* = 6), PB-NK of low-grade PC patients (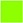 bars) (*n* = 5: Pt. 1, Pt. 6, Pt. 7, Pt. 8, Pt. 9) and PF-NK of low-grade PC patients (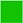 bars) (*n* = 3: Pt. 1, Pt. 6, Pt. 8) **(A)**. Cytofluorimetric analysis of the expression of a panel of cell surface markers on HD-NK (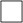 bars) (*n* = 6), PB-NK of high-grade PC patients (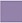 bars) (*n* = 3: Pt. 2, Pt. 3, Pt. 4) and PF-NK of high-grade PC patients (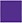 bars) (*n* = 3: Pt. 2, Pt. 3, Pt. 4) **(B)**. Cells are gated on CD56dim NK cells. In order to compare the expression of the three groups simultaneously (i.e., HD-NK, PB-NK, and PF-NK), we computed the Krustall rank sum tests for each cell surface marker analyzed. ^*^P < 0.05. **(A,B)** Dot plots derived from a representative healthy donor (HD-NK), a representative low-grade PC patient (PB-NK/PF-NK) and a representative high-grade PC patient (PB-NK/PF-NK) are shown. Percentages of positive NK cells (gated on CD56dim subset as indicated by the dotted line) for the indicated receptors are reported in the upper right quadrant of each dot plot **(C)**.

These analyses were performed using R software (Figures 1, 2).

**Figure 3:**
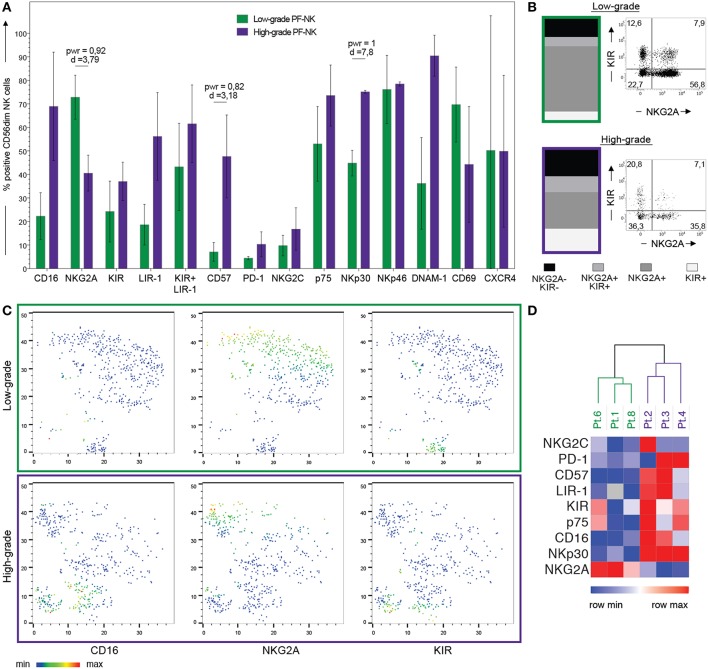
Comparison of PF-NK cells derived from low-grade and high-grade PC patients. Cytofluorimetric analysis of the expression of a panel of cell surface markers on PF-NK derived from low-grade PC patients (low-grade PF-NK) (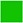 bars) (*n* = 3:Pt. 1, Pt. 6, Pt. 8) and PF-NK derived from high-grade PC patients (high-grade PF-NK) (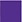 bars) (*n* = 3: Pt. 2, Pt. 3, Pt. 4). The power (pwr) and Cohen's distance (d), calculated with the Power Analysis for Two-group Independent sample *t*-test, of the pair are indicated when significant (power ≥ 0,8) **(A)**. Distribution of KIR and NKG2A receptors on gated CD56dim NK cells of low-grade PC patients and high-grade PC patients. A representative donor is shown for each group of patients **(B)**. t-SNE clusterization of CD56dim PF-NK cells derived from a representative low-grade PC patient (Pt. 6) and a representative high-grade PC patient (Pt. 4). The color scale represents the different distribution of CD16, NKG2A, and KIR receptors evaluated through the variation of Geo-mean **(C)**. Heatmap showing the clustering of low-grade (Pt. 1, Pt. 6, Pt. 8) and high-grade (Pt. 2, Pt. 3, Pt. 4) PC patients based on the differential expression of the indicated NK cell receptors on gated CD56dim NK cells. The color scale goes from blue (low relative expression) to red (high relative expression) and it is based on the variation of each single receptor through the six patients **(D)**.

In order to compare the expressions of low-grade and high-grade PF-NK, we performed the Power Analysis for Two-group Independent sample *t*-test, using the R package pwr. In this test “d” represents the *Cohen's d* that is an effect size used to indicate the standardized difference between two means. *Cohen's d* equal to 0.2, or to 0.5, or to 0.8 were considered a small, medium and large effect size. Each test was considered valid if the significance level was ≤ 0.05 and if the power was ≥ 0.80 (Figure 3).

## Results

### Phenotypic Analysis of Peripheral Blood and Peritoneal Fluid/Ascites NK Cells From Patients Affected by Peritoneal Carcinomatosis

To analyze the impact of the PC tumor microenvironment on NK cells, we characterized fresh NK cells derived from the peripheral blood (hereafter termed as PB-NK) and from the peritoneal fluid/ascites (hereafter termed as PF-NK) of patients affected by low-grade and high-grade PC by flow cytometry for the expression of a large panel of cell surface markers. The results were compared with those obtained using NK cells derived from peripheral blood of healthy donors (hereafter termed HD-NK). CD56bright NK cell subset on PF-NK cells showed phenotypic features similar to those of classical CD56bright PB-NK cells although the proportion of this subset was higher in the PF compartment (PF average score: 39%; PB average score: 9%). On the other hand, CD56dim PB-, and PF-NK cells derived from the same patients and HD-NK cells showed some differences in the expression of both activating and inhibitory NK cell receptors. In particular, CD56dim PF-NK cells displayed lower levels of KIR and CD57 (a marker typical of terminally differentiated NK cells) and higher level of PD-1 as compared to CD56dim HD- and PB-NK cells (Figure 1). Interestingly, most CD56dim PF-NK cells displayed a significant down-regulation of CD16, the Fc-gamma receptor responsible for antibody-dependent cellular cytotoxicity (ADCC), as compared to CD56dim HD- and PB-NK cells (Figure 1). Moreover, the CD56dim PF-NK cell subset showed a trend of increment in CD69 expression, thus indicating that activated NK cells represented a large fraction of PF-NK cells (Figure 1).

We also observed that the expression of CXCR4 was strongly downregulated in PB-NK cells as compared to autologous PF-NK cells as well as to HD-NK cells, suggesting a possible role of this chemokine receptor in the recruitment of CD56dim NK cells from the periphery to the tumor microenvironment. In this context, we analyzed PF and plasma from matched patients for the presence of up to 50 soluble factors using a Multiplex Assay, in order to identify mediators potentially involved in the shaping of the phenotypic/functional characteristics of PF-NK cells (Table 1). This analysis revealed that PF is generally enriched with the SDF-1alpha chemokine (the ligand for the CXCR4 receptor) as compared to plasma derived from the same patients. In some cases, this increase was >70%.

**Table 1:** Soluble factors detected in plasma and peritoneal fluid (PF) of low-grade and high-grade PC patients compared with soluble factors detected in healthy donors (HD) plasma.

**Patient**	**Sample**	**VEGF**	**TRAIL**	**TNF-b**	**TNF-a**	**SDF-1a**	**SCGF-b**	**SCF**	**RANTES**	**PDGF-bb**	**MIP-1b**	**MIP-1a**	**MIG**	**MIF**	**MCP-3**	**MCP-1 (MCAF)**	**M-CSF**
HD 1	Plasma	10.28	160.055	460.8	88.565	695.965	202488.01	32.35	ND	20667.39	258.245	1.96	2364.995	1895.435	0.22	11.435	30.97
HD 2	Plasma	199.075	138.935	396.9	111.785	789.445	124500.015	52.735	ND	23018.27	280.365	1.17	339.63	2471.425	0	24.26	58.335
Pt. 1	Plasma	173.225	49.87	233.2	20.44	351.98	38700.6	6.91	2152.38	175.59	131.345	0.865	476.08	25.89	0	29.38	6.29
Pt. 2	Plasma	26.26	27.05	85.2375	2.855	113.3075	22372.235	7.2675	1001.33	52.9575	59.7825	1.01	240.6425	138.35	0.11	51.6375	1.5825
Pt. 3	Plasma	0	11.875	38.81	0.94	24.585	116900.705	7.74	296.505	84.65	59.86	1.91	822.31	194.72	1.82	580.605	13.38
Pt. 4	Plasma	182.73	101.465	289.27	29.755	471.935	61759.515	16.055	2810.5	217.905	159.76	0.85	669.645	1420.815	0	114.295	5.475
Pt. 6	Plasma	0	132.93	246.78	56.61	606.365	79799.94	69.865	6180.09	304.16	144.87	3.085	4405.87	853.22	2.415	137.95	48.335
Pt. 7	Plasma	149.805	119.5	280.45	24.905	461.575	42892.27	23.43	6357.5	748.89	172.025	0.96	665.3	940.995	0.42	96.775	19.64
Pt. 8	Plasma	0	107.385	285.975	36.42	534.265	83015.745	26.33	11953.38	403.195	169.9	1.315	418.4	1305.49	0	68.695	0
Pt. 1	PF	243.795	32.4	45.635	40.81	1312.535	204341.7	51.31	309.675	94.265	60.275	6.34	1723.75	361.75	3.515	66.62	21.815
Pt. 2	PF	0	20.395	36.855	9.275	156.17	2373.91	5.37	327.12	39.58	28.25	1.235	85.095	1631.79	0	19.845	0.88
Pt. 6	PF	206.17	161.195	70.05	272.79	1939.845	180303.03	71.175	75.25	170.66	244.47	133.45	10117.56	17280.6	454.985	ND	41.475
Pt. 8	PF	131.145	25.785	39.775	57.16	1165.935	76851.885	38.05	171.555	56.12	88.06	12.32	488.88	5486.46	6.9	145.96	4.87
**Patient**	**Sample**	**LIF**	**IP-10**	**IL-9**	**IL-8**	**IL-7**	**IL-6**	**IL-5**	**IL-4**	**IL-3**	**IL-2Ra**	**IL-2**	**IL-1ra**	**IL-1b**	**IL-1a**	**IL-18**	**IL-17**
HD 1	Plasma	4.23	1918.885	421.4	233.155	51.89	2.21	0	4.74	0	118.125	0	426.365	2.12	9.32	101.44	4.71
HD 2	Plasma	3.14	2095.385	352.525	97.61	146.895	6.38	0	9.055	0	69.145	0	1751.08	6.075	8.505	100.32	14.895
Pt. 1	Plasma	13.995	400.91	223.48	4.66	12.615	0.955	0	2.265	0	43	0	0	0.645	5.435	9.695	0
Pt. 2	Plasma	5.5275	186.65	88.1125	7.0725	2.26	0.53	0	1.405	0	23.8325	0	33.89	0.635	2.4325	7.44	0
Pt. 3	Plasma	6.375	0	34.63	60.44	0	277.05	0	1.025	0	60.31	0	259.99	0.71	4.865	18.775	0
Pt. 4	Plasma	0	788.26	266.22	50.34	0.605	21.285	0	4.47	0	45.6	0	375.455	1.11	0	13.435	0
Pt. 6	Plasma	0.58	1033.445	222.565	19.42	0	19.555	7.855	2.79	0	539.595	0	127.415	0.825	8.28	98.205	1.065
Pt. 7	Plasma	0	93.41	296.04	33.58	36.3	25.775	8.875	1.635	0	51.59	0	31.2	0.8	4.865	14.865	0
Pt. 8	Plasma	110.9	440.87	262.735	14.55	9.07	0.845	0	1.205	0	81.025	0	116.585	0.075	1.035	18.46	0
Pt. 1	PF	30.495	5694.53	42.955	118.26	7.73	657.55	9.18	2.405	0	509.645	1.24	39.36	0.835	9.725	14.21	3.96
Pt. 2	PF	6.17	221.97	42.48	6.35	2.645	1.87	0	0.8	0	8.215	0	51.89	0.295	1.215	2.065	1.44
Pt. 6	PF	117.065	#DIV/0!	74.45	9170.92	15.59	5494.13	55.24	11.35	1.17	1067.33	6.665	4488.69	4.62	27.755	73.68	22.25
Pt. 8	PF	60.945	1760.75	41.615	395.75	15.505	649.12	11.445	3.63	0	252.995	0.105	166.42	0.72	3.72	16.01	6.125
**Patient**	**Sample**	**IL-16**	**IL-15**	**IL-13**	**IL-12p40**	**IL-12(p70)**	**IL-10**	**IFN-g**	**IFN-a2**	**HGF**	**GROa**	**GM-CSF**	**G-CSF**	**FGF b**	**Eotaxin**	**CTACK**	**b-NGF**
HD 1	Plasma	327.035	6.97	0	33.205	0.07	1.465	0	3.02	2074.565	526.38	0	48.84	8.225	40.745	1716.99	1.345
HD 2	Plasma	908.425	15.375	0	30.5	0	1.465	40.335	2.33	2042.815	952.65	5.325	113.035	13.66	405.53	955.815	3.4
Pt. 1	Plasma	29.68	12.625	0	2.65	0.07	0.02	0	0	238.33	299.14	0	50.745	0.825	83.525	989.48	1.345
Pt. 2	Plasma	34.69	0	0	3.0775	0	1.245	0	0	180.8	123.745	0	33.79	1.2375	31.3125	465.12	0
Pt. 3	Plasma	6.45	0	0	12.35	0.07	0	125.35	4.74	2187.025	0	0	54.435	2.475	21.865	79.455	0
Pt. 4	Plasma	55.1	0	0	0	0.88	0	8.825	0	424.265	317.125	0	95.745	2.475	72.93	1791.72	0
Pt. 6	Plasma	171.58	329.125	0	3.57	0.07	0	91.275	1.585	902.7	389.41	5.705	114.815	4.265	133.575	1452.625	5.895
Pt. 7	Plasma	81.985	194.27	0	11.07	2.985	273.685	0	0	443.885	443.59	4.31	65.075	3.645	129.18	755.355	9.065
Pt. 8	Plasma	84.785	0	0	0	0	1.465	6.32	0.955	612.41	290.065	0	59.03	0.825	134.885	924.92	0
Pt. 1	PF	219.89	3.39	0.145	60.135	0.59	22.765	78.11	3.7	3570.565	125.21	0	145.975	5.49	15.27	1370.125	5.345
Pt. 2	PF	62.965	0	0	4.51	0.02	0.54	0	1.495	148.58	54.315	0	23.845	41.905	8.92	115.165	0.175
Pt. 6	PF	867.895	48.965	3.055	93.975	1.715	28.525	441.335	22.48	2151.51	622.24	3.625	1747.37	13.415	59.71	1081.29	7.54
Pt. 8	PF	224.455	0	1.07	51.775	0.22	69.325	54.42	5.105	2120.91	195.885	0.315	273.885	15.695	58.47	1013.39	6.225

### Phenotypic Comparison Between PB- and PF-NK Cells Derived From Low-Grade or High-Grade PC Patients

To understand if the differences between PB- and PF-NK cells were features common to all patients or were dependent on the tumor grade, we divided the patients into two groups: one characterized by a low-grade disease (which included the well-differentiated mesothelioma and the low-grade pseudomyxoma) and the other characterized by high-grade disease (which included peritoneal metastasis of primary colon or ovarian carcinomas). By comparing the PB- and PF-NK cells of low-grade PC patients, many differences highlighted in Figure 1 were confirmed and even more evident. In particular, we observed that the low-grade PC microenvironment was enriched with more immature NK cells, characterized by lower expression of CD16, KIR, LIR-1, and CD57 and higher expression of NKG2A, as compared to autologous PB (Figures 2A,C and Supplementary Figures 1, 2). In this regard, it is important to consider that a CD16 downregulation was previously described also on PF-NK cells from ovarian cancer patients, mainly due to soluble factors, including IL-18 and TGF-beta (34, 35), present in this compartment. In addition, a distinct CD56dim NK cell subset, characterized by lower expression of CD16 and KIR and undetectable levels of CD57, was described by different research groups in different pathological conditions (39–41). Moreover, the CD56dimCD16dim NK cell subset expressed higher levels of CXCR4 (39).

In our case, the CD56dim PF-NK cell subset of low-grade patients is characterized by the same features of the subset previously described, including higher levels of CXCR4 as compared to autologous PB (Figure 2 and Supplementary Figures 1, 2).

On the other hand, no significant differences were detectable between PB- and PF-NK cells from high-grade PC patients (Figures 2B,C and Supplementary Figures 1, 2).

### Comparison of PF-NK Cells Derived From Low-Grade and High-Grade PC Patients

To fully understand if, in low-grade and high-grade PC microenvironment, distinct features characterize NK cells, we performed further analyses comparing PF-NK cells derived from low-grade or high-grade PC.

Interestingly, in the PF of low-grade PC patients a large fraction of CD56dim NK cells showed features of immature NK cells, characterized by the NKG2A+KIR-CD57-CD16dim phenotype, whereas the percentage of CD56dim NK cells expressing KIR (Figures 3A,B) and LIR-1 (Figure 3A) was increased in the PF of high-grade PC patients. In addition, PF of low-grade PC patients contained a substantially higher percentage of CD56bright NK cells than PF of high-grade PC patients (average score: 48 vs. 27%).

By using the t-SNE algorithm, we observed that the co-expression pattern of markers on NK cells generated specific clusters associated with selected NK cell receptors (CD16, NKG2A, and KIR), in the PF-NK cells of low-grade and high-grade PC patients (Figure 3C). Furthermore, this approach allowed to better visualize the different distribution of these NK cell receptors in the PF-NK cells of these two groups of patients.

**Figure 4:**
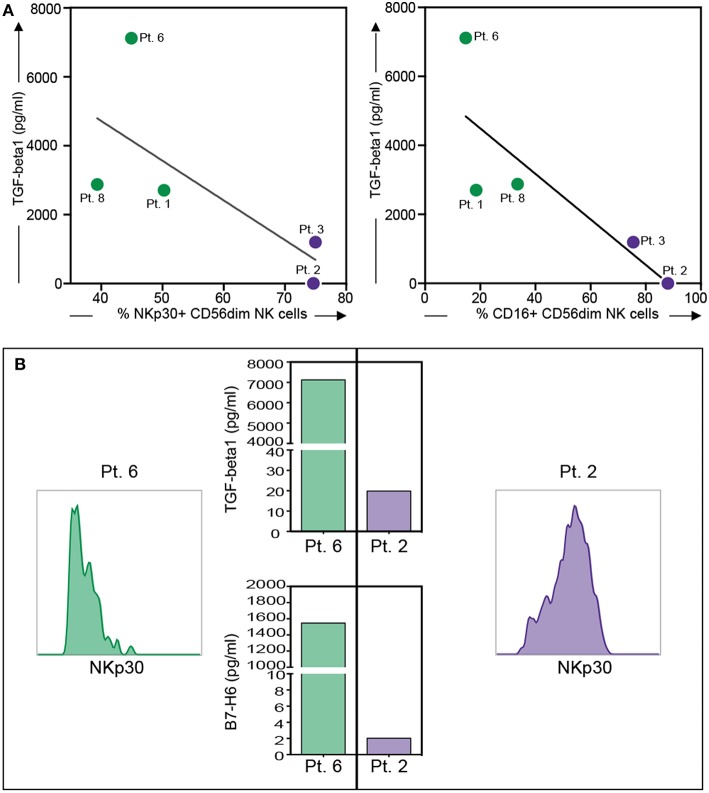
Relationship between NKp30 or CD16 expression on PF-NK cells and soluble factor's concentration in the PF of low-grade and high-grade PC patients. Relationship between NKp30 or CD16 receptor downregulation on PF-NK cells and TGF-beta1 concentration in the PF of low-grade PC patients (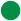 dots, *n* = 3: Pt. 1, Pt. 6, Pt. 8) or high-grade PC patients (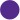 dots, *n* = 2: Pt. 2, Pt.3). The linear regression line is shown (NKp30 vs. TGF-beta1: *R* = 0.52 and *p* = 0.17; CD16 vs. TGF-beta1: *R* = 0.68 and *p* = 0.09) **(A)**. Histograms showing the expression of NKp30 on PF-NK cells of a representative low-grade PC patient (Pt. 6) and a representative high-grade PC patient (Pt. 2) and TGF-beta1/sB7-H6 concentration in PF of the same patients **(B)**.

Moreover, an impaired expression of some activating NK receptors, primarily NKp30 and DNAM-1, was shown on the PF-NK cells from low-grade PC patients (Figure 3A). Previous studies demonstrated that the defective expression and function of activating NK cell receptors, including NKp30 and/or DNAM-1, may be induced by the chronic engagement of these receptors by their ligands in soluble form or expressed on tumor cells. In this context, it is important to consider that CD45neg primary cell lines derived from PF expressed some activating NK receptor ligands including PVR and Nectin-2 (DNAM-1 ligands) (35), and B7-H6 (NKp30 ligand) (34), although, in some of the cases analyzed, this last molecule was only detectable in the cytoplasm and not at the cell surface (Supplementary Figure 3). In addition, PF microenvironment of low-grade PC patients contained a much higher concentration of TGF-beta1 (an increment over 80%) as compared to high-grade PC patients. Considering that TGF-beta1 is known to induce down-regulation of some activating NK cell receptors, including NKp30 (42), it is likely that the downmodulation of NKp30 observed in low-grade PC patients may be the result of the combined action of soluble B7-H6 (sB7-H6) and TGF-beta1. Further analysis indicated that a relationship between the level of NKp30 downmodulation on PF-NK cells and the concentration of TGF-beta1 and sB7-H6 in the PF could be established. More specifically, low-grade PC patients displaying an important decrease in the expression of NKp30 exhibited also a high concentration of TGF-beta1 and sB7-H6 in the PF. On the other hand, high-grade patients displaying higher levels of surface NKp30 on PF-NK were characterized by very low concentration of TGF-beta1 and sB7-H6 in the PF (Figures 4A,B).

Notably, TGF-beta1 could also be involved in the CD16 impairment (43, 44) detected in the PF-NK of low-grade PC patients and in the induction of NKG2A expression, as previously described in other tumor types (45, 46). Indeed, it was possible to observe a relationship between the level of CD16 downmodulation on PF-NK cells and TGF-beta1 concentration in the PF (Figure 4A).

Thus, the impaired expression of both activating and inhibitory receptors on PF-NK cells from low-grade PC patients induced by tumor cells and/or soluble factors present in the PF microenvironment may contribute to the development of escape mechanisms from immune surveillance.

Regarding the PF-NK cells from high-grade PC patients, we observed a trend of increment in the expression of the PD-1 inhibitory checkpoint. The fact that this difference is not clearly significant may depend on the small number of PC patient samples analyzed, due to the rarity of this disease. Considering that the expression of this inhibitory receptor on NK cells can compromise their anti-tumor activity, and that PF-derived primary cell lines express both PD-L1 and PD-L2 (Supplementary Figure 3), the increased PD-1 expression may play a role in inducing NK cell impairment in tumor control.

To support our findings regarding the significant differences between PF-NK cells derived from low-grade and high-grade PC patients, we perform hierarchical clustering in order to verify the presence of particular patterns in our data. In Figure 3D, the two-color heatmap plots show the unsupervised hierarchical clustering of NK samples derived from PF of PC patients. This analysis clearly separates low-grade from high-grade samples, based on the different expression of a small group of classical NK cell molecules, including the activating and inhibitory receptors appointed above.

Thus, the low-grade or high-grade PC tumor microenvironment may differently contribute to the tumor escape from immune surveillance, by inducing different shaping in the tumor-associated NK cell receptors repertoire (e.g., down-regulation of activating receptors vs. up-regulation of inhibitory ones).

### Functional Comparison Between PB- and PF-NK Cells Derived From Low-Grade or High-Grade PC Patients

To support our results regarding the impaired expression of some activating NK receptors (primarily NKp30) on the PF-NK cells from low-grade PC patients, degranulation assays against K562, a HLA-I negative cell line previously shown to express B7-H6, were performed in the absence or in the presence of the anti-NKp30 blocking mAb. The results were compared with those obtained using HD-NK cells.

**Figure 5:**
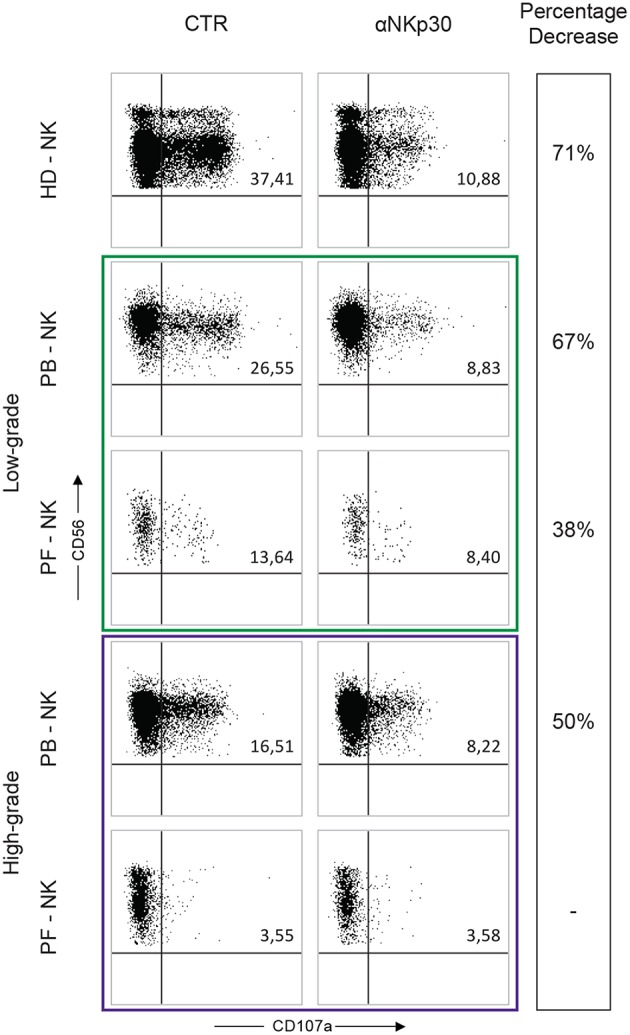
Degranulation assay of PB- and PF-NK cells derived from low-grade and high-grade PC patients. Degranulation assay (CD107a expression) of PB- and PF-NK cells from a representative low-grade PC patient (Pt. 6), a representative high-grade PC patient (Pt. 3) and a representative HD stimulated by K562 tumor target cells, in the absence or in the presence of anti-NKp30 mAb. Percentages of CD107a+ NK cells are reported in the upper right quadrant of each dot plot.

As shown in Figure 5, PB-NK cells derived from a representative low-grade PC patient or a representative high-grade PC patient as well as HD-NK cells displayed ability to degranulate following exposure to K562. These functional activities were inhibited in the presence of anti-NKp30 mAb, capable of disrupting the NKp30/B7-H6 interaction.

On the contrary, under the same conditions, PF-NK cells derived from both types of PC patients were characterized by substantial differences in terms of degranulation. In particular, as expected, the degranulation of PF-NK cells derived from the low-grade PC patient (characterized by a compromised expression of NKp30) was lower than that of HD-NK and autologous PB-NK cells (expressing normal levels of NKp30). Again the degranulation was partially inhibited in the presence of anti-NKp30 mAb.

On the other hand, unexpectedly, the degranulation activity of PF-NK cells derived from the high-grade PC patient was deeply impaired, either in the absence or in the presence of anti-NKp30 (Figure 5), despite the normal expression of NKp30. The same result was obtained by using PD-L+ tumor cell lines (including OVCAR5), either in the absence or in the presence of anti-PD-1 mAb (not shown). These data suggest that PF-NK cells derived from the high-grade PC patient present additional impairments (other than PD-1-mediated block) still to be defined. In this context, it is important to consider that in the PF the ratio between NK cells and tumor cells is clearly unbalanced toward tumor cells, probably due to a defective migration of NK cells within this compartment. This event can decrease the chances that NK cells can efficiently meet and kill cancer cells even in *in vitro* experiments.

### Impact of a CRS/HIPEC Successful Treatment on NK Cell Compartment: A Case Presentation

**Figure 6:**
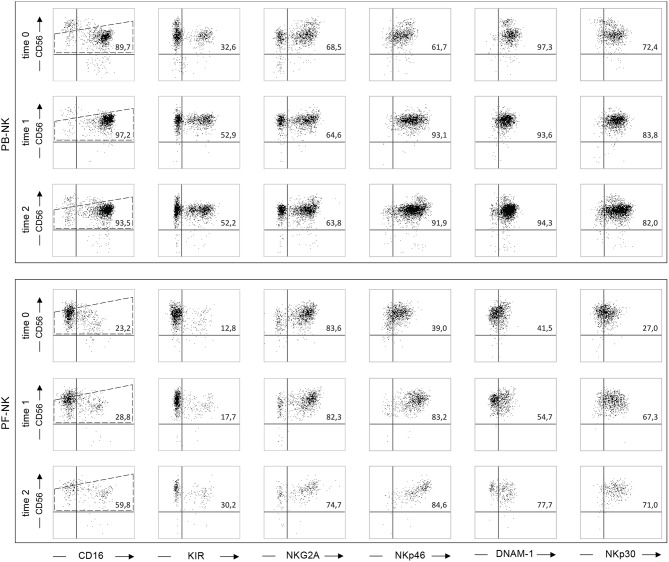
Reversion of the altered NK cell phenotype in a low-grade PC patient after the CRS/HIPEC treatment. Dot plots illustrating the expression of different NK cell receptors on PB-NK and PF-NK derived from a low-grade PC patient (Pt. 1), before treatment (time 0), and 48 h (time 1), or 7 days (time 2) after CRS/HIPEC treatment. Percentages of NK cells (gated on CD56dim subset as indicated by the dotted line) positive for the indicated receptors are reported in the upper right quadrant of each dot plot.

In order to acquire insights on the effect of the CRS/HIPEC therapy, we characterized PB- and PF-NK cells derived from Patient 1, affected by pseudomyxoma peritonei, before (time 0), and at different time points (time 1 and time 2) after therapy. At time 0, PF-NK cells, but not PB-NK cells, showed a highly compromised phenotype in terms of anti-tumor potential. In fact, PF-NK cells were characterized by a strong down-regulation of the main NK cell-activating receptors, in particular NKp46, DNAM-1, NKp30, and CD16 (Figure 6). In addition, differently from autologous PB, a higher fraction of PF-NK cells was NKG2A+, whereas only a minor cell subset expressed KIR. Notably, starting from time 1, the percentages and surface density of NKp46 and NKp30 were greatly increased, whereas those of DNAM-1 were only slightly increased. At time 2, the expression of all activating receptors, including CD16, was almost completely recovered and comparable to that of the patient's PB (and to a HD-NK, see Figures 1, 2A,C). Furthermore, a re-balancing in the distribution of KIR and NKG2A could be observed. In fact, if at time 0 almost all PF-NK cells were NKG2A+ KIR-, at time 2 the percentages of KIR+ NK cells were increased by 60% and the percentages of NKG2A+ NK cells were decreased by 25%. This result means that the CRS/HIPEC therapy in PC tumor patients can induce phenotypic changes on NK cells, thus reverting tumor-induced NK cell suppression, although the molecular mechanisms of this effect still need to be fully clarified. Importantly, the prognosis of patient 1 was very favorable. To date 4 years after treatment, this patient is in good health, with no evidence of disease recurrence.

## Discussion

Peritoneal carcinomatosis (PC) is the site of histologically heterogeneous rare primitive diseases (including pseudomixoma peritonei and mesothelioma) and one of the problematic sites of metastases for abdominal malignancies, including gastrointestinal, and ovarian cancers (1–4). Its presentation is usually associated with a significantly reduced quality of life and a very poor prognosis. To date, PC remains among the most common causes of death from abdominal cancers.

Intraperitoneal chemotherapy with surgical debulking is associated with higher survival rates than systemic chemotherapy, but results are still disappointing, and treatment is rarely curative (3). Several independent groups have reported that immune modulating agents can provide a significant therapeutic benefit in preclinical models of PC (47–50). These promising results suggest that strategies based on increasing the anti-tumor immune response within the peritoneal cavity should be pursued in PC treatment.

NK cells are a major component of the anti-tumor immune response and are involved in controlling tumor progression and metastases. However, tumors have evolved mechanisms of immunoevasion, including production of pro-tumoral/immunosuppressive cytokines, expression of ligands for inhibitory receptors, or loss of ligands for activating receptors in order to escape from the NK cell-mediated attack. Here, we show that in PC patients, tumor-associated NK cells are characterized by phenotypic and functional dysfunctions. Interestingly, the NK cell impairment is different between patients with low-grade or high-grade PC. In particular, we observed a large fraction of immature NKG2A+ CD57- NK cells displaying a strong downregulation of the main activating NK cell receptors (such as NKp30, DNAM-1, and CD16) in low-grade PC patients vs. a most mature KIR+ CD57+ NK cell fraction showing an up-regulation/induction of the immune checkpoint PD-1 in high-grade PC patients. In both cases, PF-NK cells displayed functional defects, however, while in low-grade PC patients these defects reflected the compromised phenotype, in the high-grade PC patients PF-NK cells showed much more important defects that only partially reflected the compromised phenotype detected. This suggests that further impairments, still to be highlighted, exist at the level of this NK cell population.

Thus, based on the tumor grade, PC microenvironment may be characterized by structural and functional reorganization by inducing different tumor escape mechanisms from immune surveillance. In this context, it has been previously demonstrated that metastatic PC from primary colorectal cancer (pCRC) is characterized by modification in tumor microenvironment and immune cell reaction. These changes include senescence in PC tumor cells, and enhancement of pro-tumoral soluble factors, including VEGF-A and TGF-beta, that promote neovascularization in metastatic niche and tumor growth/invasion/metastasis formation, respectively. The authors also found high amount of the NK cell-regulating cytokine IL15 and a significantly increased numbers of mature (CD57+) NK cells expressing CD107a on the surface and releasing high levels of IFN-gamma and TNF (47). Another research group has recently demonstrated that recruitment of cytotoxic, IFN-gamma-secreting, NK cells is associated with reduced tumor burden, and improved survival in a colon cancer model of PC (48). All these data suggest that NK cells can play a crucial immunosurveillance role in PC. Thus, the balance between anti-tumor and pro-tumor effects can deeply influence the anti-tumor activity of these immune cells and the metastasis formation.

Understanding the mechanisms by which PC tumor microenvironment works in inducing immune cells suppression is a complex issue, also considering that these types of tumors are uncommon and histologically heterogeneous, ranging from benign to highly malignant.

By analyzing the phenotype of NK cells derived from PF of low-grade and high-grade PC patients, we found that the PF compartment of low-grade disease is enriched in most immature NKG2A+KIR-CD57-CD16dim NK cells. A NK cell subset characterized by a similar phenotype and endowed with multifunctional activity (including potent killer and IFN-gamma producing capacity) was found in the bone marrow both of healthy children and of pediatric leukemic patients and it has been suggested that this NK cell subset can represent an intermediate differentiation stage between CD56brightCD16neg/dim and CD56dimCD16bright NK cells (39). Interestingly, these immature PF-NK cells are characterized by a strong downregulation of the main activating NK cell receptors (primarily NKp30 and DNAM-1) that may be a consequence of their engagement by specific ligands expressed on tumor cell surface or present in soluble form together with immunomodulatory soluble factors in the PF. In this context, it has been previously demonstrated that chronic receptor-ligand interactions may dampen the surface expression of some activating NK receptors, thus affecting the ability of NK cells to kill tumor cells expressing ligands for those receptors. In particular, a soluble form of B7-H6 (sB7-H6), the main NKp30 ligand, was found in the PF-microenvironment of ovarian cancer patients (34). A high amount of sB7-H6 is correlated with a greater downmodulation of NKp30. These NK cells display impaired IFN-gamma production and cytolytic function, thereby showing poor NK cell-mediated elimination of B7-H6+ ovarian cancer cells (34). Moreover, the activating NK cell receptor DNAM-1 can also be downmodulated by the chronic exposure to the ligand expressed on the surface of ovarian tumor cells (35). In addition, TGF-beta can also contribute to the downmodulation of the NKp30 activating receptor (42). Consistent with these observations, we detected the expression of ligands for NKp30 (B7-H6) and DNAM-1 (PVR, Nectin-2) on PF-derived tumor cells and several soluble factors, including sB7-H6, and TGF-beta, in the PF. These factors are certainly involved in the induction of the compromised phenotype observed in the PF-NK cells of low-grade PC patients.

With disease progression (high-grade PC tumors), we found that expression of activating NK cell receptors (including NKp30, DNAM-1, and CD16) was recovered, and the expression of inhibitory receptors (such as KIRs, LIR-1, and PD-1) increased together with the maturation state of NK cells.

Thus, low-grade and high-grade PC tumors shape their environment differently in order to evade NK cell anti-tumor immunity. Along this line, PF-NK cells from both low-grade and high-grade PC patients showed decreased NK cell degranulation function. Importantly, this impairment was apparently more pronounced in NK cells from high-grade PC patients, even if, in this case, it is not clear which mechanisms lead to the lack of NK cell degranulation capacity. Indeed, this defect also occurs with respect to target cells (i.e., K562) that do not express ligands for inhibitory receptors (i.e., HLA-I and PD-Ls molecules) up-regulated on PF-NK cells, but express ligands for activating receptors (e.g., B7-H6) that are normally expressed on these cells. However, because the percentage of NK cells was severely reduced in the PF of the high-grade PC, the fact that PF-NK cells from high-grade PC tumor were unable to fulfill their degranulation function is not surprising considering their low proportion within the tumor. Further investigations will be necessary to clarify this aspect.

Thus, cancer cells and the tumor milieu can deeply alter the functional composition of anti-tumor effector cells, including cytotoxic NK cells. This consideration highlights the importance of developing future therapies able to restore NK cell cytotoxicity and limit/prevent tumor escape from anti-tumor immunity.

Remarkably, we show that in a low-grade PC patient, with no evidence of disease recurrence after 4 years after treatment, CRS/HIPEC treatment could restore the NK cells impaired phenotype, thus reverting tumor-induced NK cell suppression. In this scenario, we can hypothesize that removal of the tumor (CRS) combined with HIPEC procedure may improve survival in low-grade PC patients by reducing the tumor factors involved in immune cell suppression (37, 51).

On the other hand, since most high-grade PC remains refractory or responds only partially to the CRS/HIPEC treatment, new therapeutic approaches aimed at increasing the effectiveness of the current therapy are necessary (52, 53). For example, immunotherapeutic reagents capable of blocking inhibitory receptors expressed by NK cells or strengthening the ADCC mechanism (mainly in high-grade PC patients with normal CD16 expression) could be associated to the CRS/HIPEC treatment in order to enhance the anti-tumor response.

In summary, this study contributes toward identification of possible markers of prognosis or candidates that may represent possible targets in immunotherapeutic approaches aiming to strengthen the anti-tumor activity of NK cells in PC tumors (15–18, 54, 55), a disease that has not been adequately investigated yet due to its rarity.

## Data Availability

The datasets generated for this study are available on request to the corresponding author.

## Ethics Statement

This study was carried out in accordance with the recommendations of the ethical standards of the institutional and/or national research committee. The protocol was approved by the ethics committee of the Liguria Region, Genova, Italy (no. 428Reg2014 for PC patients and no. 39/2012 for healthy donors). All subjects gave written informed consent in accordance with the Declaration of Helsinki.

## Author Contributions

All authors listed have made a substantial, direct and intellectual contribution to the work, and approved it for publication.

### Conflict of Interest Statement

The authors declare that the research was conducted in the absence of any commercial or financial relationships that could be construed as a potential conflict of interest.
