# Supplementary material for: Different Features of Tumor-Associated NK Cells in Patients With Low-Grade or High-Grade Peritoneal Carcinomatosis
Source: Front Immunol. 2019 Aug 21;10:1963. doi: 10.3389/fimmu.2019.01963 (PMC6712073; doi:10.3389/fimmu.2019.01963)
Supplement: Supplementary file 1 [file Image_1.pdf]

Supplemental Figure 1

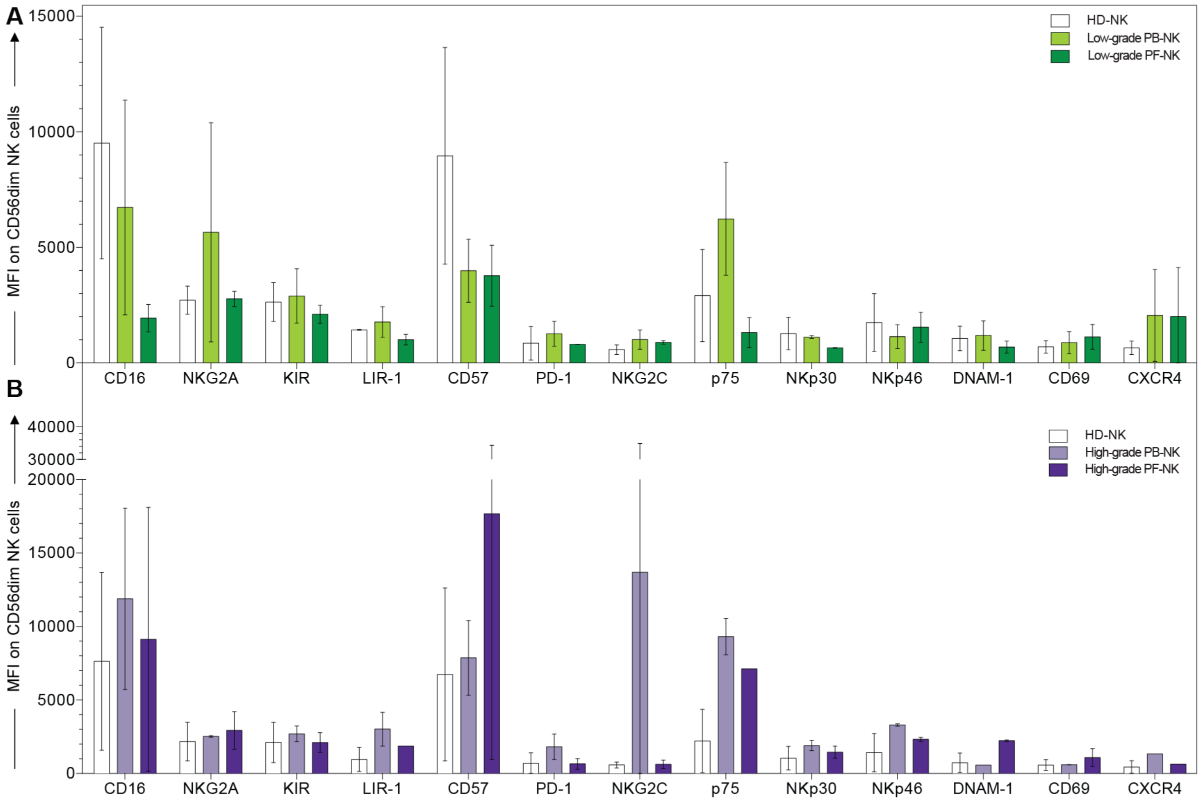

Comparison of MFI of main NK cell markers on PB- and PF-NK cells derived from low-grade and high-grade PC patients.

Mean Fluorescent Intensity (MFI) of a panel of cell surface markers on HD-NK (□ bars) (n=6), PB-NK of low-grade PC patients (■ bars) (n=5: Pt. 1, Pt. 6, Pt. 7, Pt. 8, Pt. 9) and PF-NK of low-grade PC patients (■ bars) (n=3: Pt. 1, Pt. 6, Pt. 8) (**A**).

MFI of a panel of cell surface markers on HD-NK (□ bars) (n=6), PB-NK of high-grade PC patients (■ bars) (n=3: Pt. 2, Pt. 3, Pt. 4) and PF-NK of high-grade PC patients (■ bars) (n=3: Pt. 2, Pt. 3, Pt. 4) (**B**). Cells are gated on CD56dim NK cells (**A** and **B**).
