# Supplementary material for: Different Features of Tumor-Associated NK Cells in Patients With Low-Grade or High-Grade Peritoneal Carcinomatosis
Source: Front Immunol. 2019 Aug 21;10:1963. doi: 10.3389/fimmu.2019.01963 (PMC6712073; doi:10.3389/fimmu.2019.01963)
Supplement: Supplementary file 2 [file Image_2.pdf]

Supplementary Figure 2

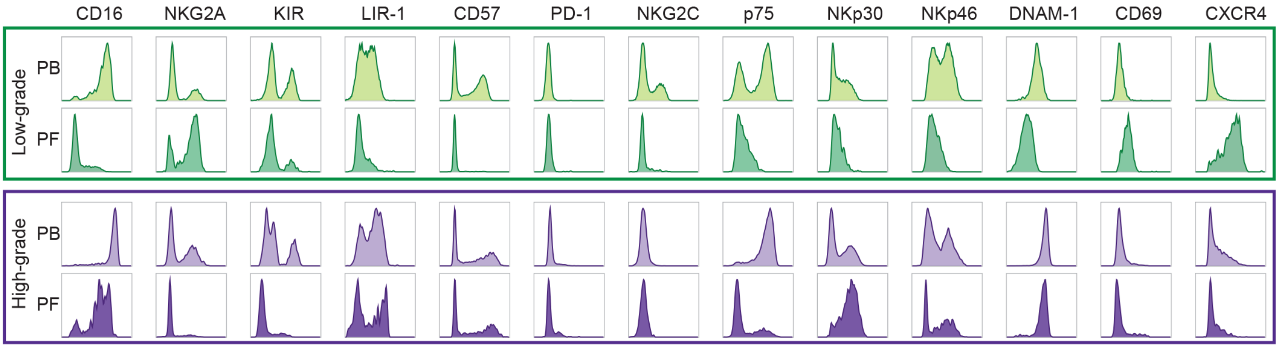

Histogram plots showing the expression of a panel of cell surface markers on PB- and PF-NK cells derived from low-grade and high-grade PC patients.

A representative low-grade PC patient (Pt. 6) and a representative high-grade PC patient (Pt. 2) are shown.
