# Supplementary material for: Different Features of Tumor-Associated NK Cells in Patients With Low-Grade or High-Grade Peritoneal Carcinomatosis
Source: Front Immunol. 2019 Aug 21;10:1963. doi: 10.3389/fimmu.2019.01963 (PMC6712073; doi:10.3389/fimmu.2019.01963)
Supplement: Supplementary file 3 [file Image_3.pdf]

Supplementary Figure 3

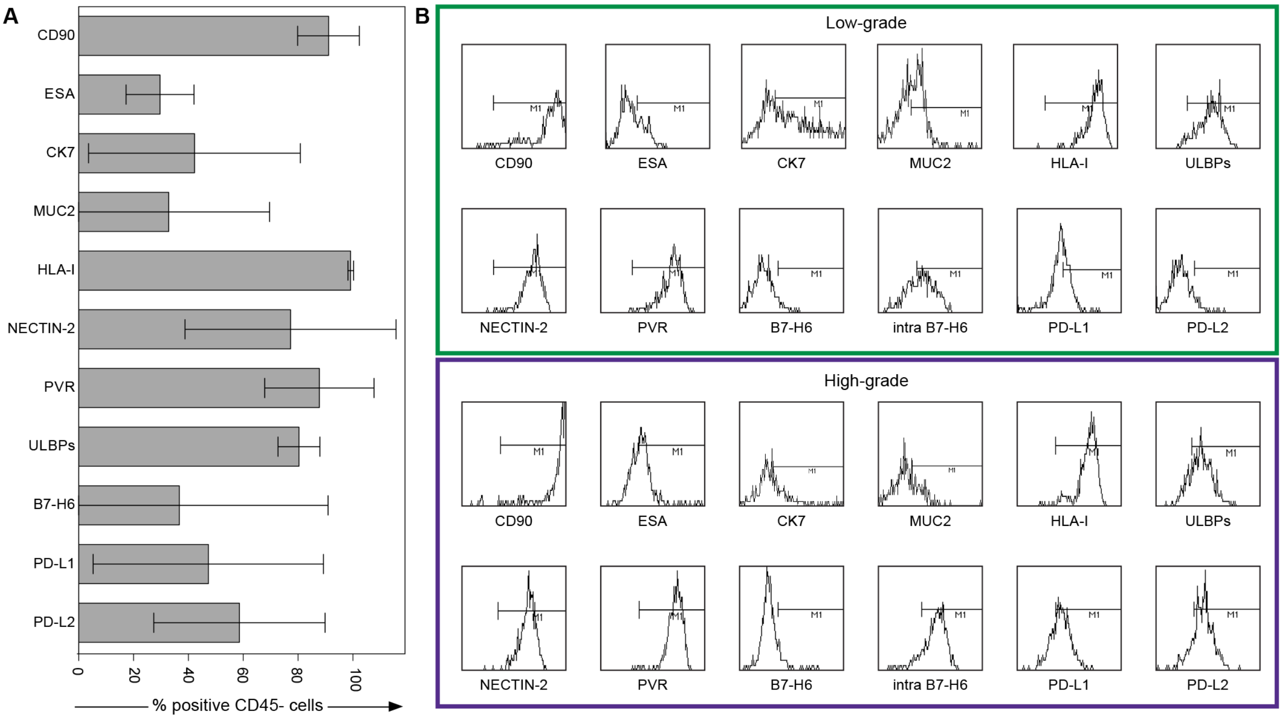

Phenotypic analysis of CD45neg primary cell lines derived from PF of PC patients.

CD45neg (CD45-) primary cell lines were analyzed for the expression of specific tumor markers (such as CD90, ESA, CK7, and MUC2) and of ligands for different activating and inhibitory NK cell receptors (such as HLA-I, PVR, Nectin-2, B7-H6, ULBPs, PD-L1 and PD-L2 molecules) (**A**). Histograms indicate the percentage of CD45neg cells positive for the indicated molecules (n=4: Pt. 1, Pt. 3, Pt. 6, Pt. 7). A representative low-grade PC patient (Pt. 1) and a representative high-grade PC patient (Pt. 3) are shown (**B**).
